# Effect of Relative Humidity and Air Temperature on the Results Obtained from Low-Cost Gas Sensors for Ambient Air Quality Measurements

**DOI:** 10.3390/s20185175

**Published:** 2020-09-10

**Authors:** Abdul Samad, Daniel Ricardo Obando Nuñez, Grecia Carolina Solis Castillo, Bernd Laquai, Ulrich Vogt

**Affiliations:** Institute of Combustion and Power Plant Technology (IFK), Department of Air Quality Control, University of Stuttgart, Pfaffenwaldring 23, 70569 Stuttgart, Germany; st158199@stud.uni-stuttgart.de (D.R.O.N.); grecia-carolina.solis-castillo@ifk.uni-stuttgart.de (G.C.S.C.); bernd.laquai@ifk.uni-stuttgart.de (B.L.); ulrich.vogt@ifk.uni-stuttgart.de (U.V.)

**Keywords:** low-cost sensors, gas sensors, air quality sensors, electrochemical low-cost sensors, air pollutants, air quality monitoring

## Abstract

Using low-cost gas sensors for air quality monitoring promises cost effective and convenient measurement systems. Nevertheless, the results obtained have a questionable quality due to different factors that can affect sensor performance. The most discussed ones are relative humidity and air temperature. This investigation aimed to assess the behavior of B4-series low-cost gas sensors from Alphasense for measuring CO, NO, NO_2_, and O_3_ for different levels of relative humidity and temperature. These low-cost gas sensors were tested for six relative humidity levels from 10% to 85% with increasing steps of 15% and four temperature levels of 10 °C, 25 °C, 35 °C, and 45 °C against reference instruments in the laboratory. The effect of these parameters on low-cost gas sensors was quantified in laboratory from which a correction algorithm was calculated, which was then applied to the field data. The applied algorithm improved the data quality of the low-cost gas sensors in most of the cases. Additionally, a low-cost dryer was assessed to reduce the influence of these factors on the low-cost gas sensors, which also proved to be suitable to enhance the data quality.

## 1. Introduction

### 1.1. Air Quality and Low-Cost Sensors

Outdoor air pollution or ambient pollution is linked to different diseases and symptoms in the human respiratory, cardiovascular, nervous, urinary, and digestive systems [1], which lead to around 4.2 million premature deaths yearly around the world [2]. Nonetheless, the poor air quality not only affects humans, but, according to research, flora and fauna are also highly disturbed [3]. In this context, many cities around the world have implemented stationary air quality monitoring stations in urban and industrial places with the purpose of knowing the state of air quality in these areas. Such monitoring stations are a valuable source of information to create and implement measures to counteract these problems. However, the representativeness of the data measured by monitoring stations needs to be assessed [4]. Consequently, there is a need of creating a wide and detailed air quality map to provide a better spatial resolution of the pollutant distribution over entire cities.

To achieve that goal, it is necessary to deploy additional monitoring stations to get higher spatial resolution of air pollutants as well as cover new locations where air quality needs to be measured. This is costly, requires constant maintenance and calibration carried out by highly skilled professionals, as well as a suitable location due to their size [5,6,7,8,9]. Therefore, it is of great importance to employ alternative equipment that is small, easy to use, accurate and especially cost-effective in order to distribute many of them in the cities providing enough information to create a consistent wide pollutant distribution map. In order to accomplish these requirements, the air quality low-cost sensors were assessed.

### 1.2. Electrochemical Gas Sensors

Recently low-cost gas sensors are being used for the application of monitoring air quality. Different types of low-cost gas sensors are available commercially [10]. The most common ones however are the electrochemical sensors. These sensors can measure the concentrations of different gases such as NO, NO_2_, O_3_, CO, and many other gases. They have good sensitivity from mg/m^3^ to µg/m^3^ and fast response time from 30 to 200 seconds [11]. The working principle consists of molecules of the target gas first diffusing through the anti-condensation membrane. Then through a capillary, filter, and hydrophobic membrane in order to reach the electrode, which gets reduced or oxidized, consuming or producing electrons, respectively, generating an electric current. The intensity of this electric current defines the concentration of the measured component [12,13]. 

For the current research, four gases were considered, namely NO, NO_2_, O_3_, and CO. A market survey was performed in order to gain information about the low-cost sensors available to measure these gases. The specifications of some of the low-cost gas sensors that are available in the market are summarized in the Appendix A (Table A1, Table A2, Table A3 and Table A4).

According to several studies carried out [11,12,14,15,16,17], the data collected by the low-cost sensors is in many cases questionable and has low quality as compared to the conventional monitoring stations. These studies have shown that the low-cost sensors are influenced not only by several external parameters such as air temperature, relative humidity and interferences with other compounds (cross sensitivity), but also by characteristics directly related to the specific low-cost sensor such as warming up time, gain of the electrical signal and initial manufacturer calibration.

Moreover, other researchers claim that relative humidity has more influence on the CO low-cost gas sensor performance and less influence on the NO low-cost gas sensor [18]. Furthermore, low-cost sensors have relatively short lifetime of around 1 to 2 years [19]. Although the low-cost sensors are to be tested under several established conditions and compared to reference instruments that can be found in conventional monitoring stations [11], there is lack of uniform guidelines, protocols or standards for the application of this new technology for regulatory purposes [14]. Therefore, by quantifying the parameters affecting the low-cost sensor results, it is expected that this new technology could serve as a support and extension of the current technology available.

### 1.3. Previous Studies

During the last years, different researchers have carried out several studies regarding low-cost air quality sensors. In general, the researchers have used mainly two techniques to study the low-cost sensors and analyze the influences from different parameters. One of this is characterization in laboratory where the desired conditions under which the low-cost sensors are to be tested are controlled and the corrections were developed for those specific conditions [15,18,20,21,22,23,24]. The advantage of this technique is that wide range of concentrations of target and co-pollutants, relative humidity, temperature and further parameters can be achieved in a precise manner. However, the behavior of the low-cost sensors changed from laboratory to field environment due to certain interferences that were not evaluated in the laboratory. Hence, the quality of the measurement was affected.

Some researchers used the second technique that consisted on the deployment of the low-cost sensors near reference monitoring stations or professional measuring instruments. These colocation measurements helped to compare and calibrate the low-cost sensors according to the data obtained from reference instruments [25,26,27,28,29]. In this case, the advantage is that the low-cost sensors were exposed directly to the desired environment in which they are to be deployed. Nevertheless, a smaller range of the evaluated parameters can be utilized for the calculation of the correction algorithms and the calibrated sensors were restricted to be used in the specific area in which they were calibrated. Machine learning can also be used in this case to remove outlier, adjust offset and improve correlation with reference instruments [30,31].

There are also studies in which the authors implemented both methods in order to take advantage of the benefits mentioned in each case. In 2016, the behavior of two commercial low-cost ozone sensors (Model OX-B421, Essex, U.K) from Alphasense was studied in laboratory, as well as field conditions by Xiao Pang et al. [22]. During the laboratory experiments, five different relative humidity levels were evaluated (15%, 45%, 60%, 75%, and 85%) using flow rate variations from 0.30 to 1 L/min. In the case of the field analysis, the values obtained during 18 days by the low-cost sensor were corrected using the data from the laboratory. The main finding was that under stable meteorological conditions, the results could be improved after applying a zeroing process to offset the baseline drifts. However, a sudden change of relative humidity (variation of 20%/min) highly affected the electrical signal of the low-cost sensor. The authors established that OX-B421 low-cost sensors are suitable to be deployed as an alternative for traditional reference devices by using a calibration curve as long as the values of relative humidity remain relatively constant.

A similar research was carried out by Xiao Pang et al. in 2018 [15], analyzing the behavior not only of an ozone low-cost sensor (OX-B431) but also CO (CO-B4), NO (NO-B4), NO_2_ (NO_2_-B42), and SO_2_ (SO_2_-B4) under different relative humidity levels. In addition, the authors checked the cross-sensitivities to different gases. Each low-cost sensor was calibrated to its target gas as well as five different co-pollutants (CO, O_3_, NO, NO_2_, CO_2_, and SO_2_) and at five relative humidity levels (15%, 45%, 60%, 75%, and 85%) maintaining a stable temperature of around 20 °C. The results obtained by the authors showed that using a simple regression correction calculated from the relative humidity values between 15% and 60% along with five different co-pollutants, the correlation values of the R^2^ were 0.83, 0.68, 0.60, and 0.75 for O_3_, CO, NO_2_, and NO low-cost sensors, respectively. The correction was applied in data obtained during 18 days field measurement. The behavior of SO_2_ low-cost sensor could not be evaluated, since the ambient SO_2_ concentrations were below the detection limit of the low-cost sensor.

Peng Wei et al. researched the impact analysis of temperature and humidity conditions on air quality low-cost sensors [18]. During this study, four low-cost sensors were tested for three relative humidity levels (17%, 30%, and 48%) as well as four air temperatures (16 °C, 19 °C, 33 °C, and 36 °C). Three correction models were formulated by the authors, which were applied to the field data collected in February 2015. The relative humidity tested in the laboratory (maximum 48%) was lower than the relative humidity measured during the field experiment, i.e., between 54% and 95%. Therefore, despite an improvement between the data obtained from the low-cost sensors and the corrected one, the authors concluded that further investigation on low-cost sensors under extreme ambient conditions is needed to cover a wider range of relative humidity and air temperature.

Additionally, Karagulian et al. [14] published a complete review of the studies that have been carried out by different institutes and universities around the world regarding this topic. They also presented a short summary of protocols and standards that are being developed by governments and research institutes to define the parameters that need to be evaluated when characterizing the behavior of these low-cost sensors. The authors stated that despite the main parameters that influence the results of the low-cost sensor measurements are known, there is a lack of accurate models that correct these effects and further investigation must be carried out in order to quantify them.

By keeping the above-mentioned investigations in mind, it was intended in this research to develop a correction algorithm that can be applied to the data obtained by the low-cost gas sensors in order to improve its quality. This algorithm should cover a wider range of the influencing parameters under investigation so that it can be implemented on the field data. In addition, it was intended to assess the behavior of the low-cost gas sensors when a low-cost dryer is implemented. The low-cost dryer could maintain the studied meteorological parameters constant in the low-cost sensor system. This could reduce the effort of developing a complex correction algorithm and get reliable values from the low-cost sensors after simple post-processing of the data.

## 2. Measurement Technique and Methodology

According to latest studies regarding low-cost gas sensors, there is a necessity to do more investigations related to the factors, such as temperature and relative humidity that can influence the data obtained by these low-cost gas sensors. To address these needs, the proposed measurement technique focused on both mentioned factors and performing experiments in laboratory and in the field (outdoor conditions). The experimental setup included sensors (electrochemical gas sensors and a sensor for measuring temperature and relative humidity), reference devices and Gas Phase Titration (GPT) system. The details of the setup are described in this section.

### 2.1. Low-Cost Gas Sensors and Reference Devices

In this research project, the 4-electrode amperometric B4-series low-cost gas sensors from the company Alphasense with their respective ISB (Individual Sensor Board) for CO, NO, NO_2_, and O_3_ gases were used. These sensors were chosen due to the fact that according to the latest studies, they are proven to be feasible to use for air quality measurements and they show good response, linearity, reliability, and repeatability [12,19,32]. The B series sensors were used since they are designed for measurements in urban air fixed site networks. More details are explained in Table 1. To compare and analyze the results obtained by the sensors, reference devices for the respective gases were used. Table 2 describes the reference devices used in this research.

Additionally, a dilution calibrator device Serinus CAL 3000 fabricated by Ecotech Company was used for the calibration of reference devices and sensors.

**Figure 1:**
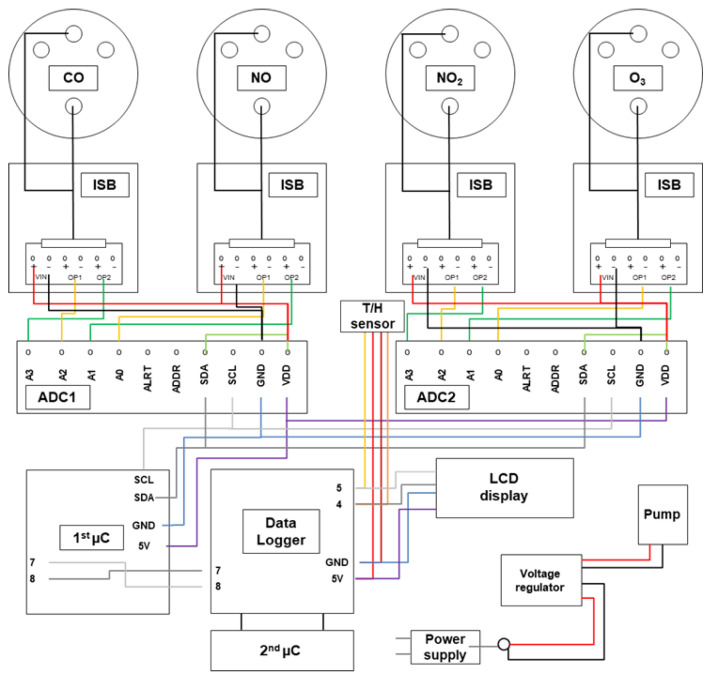
Low-cost gas sensor platform with CO, NO, NO_2_, and O_3_ sensors.

The low-cost sensor platform and its components are shown in Figure 1. The low-cost gas sensors (CO, NO, NO_2_, and O_3_) were placed in a sensor chamber and the sensors were connected to their respective sensor boards (ISB) along with a main board for power supply, two analog-to-digital converters (ADC) and data recording (Data Logger). Additionally, a temperature and relative humidity sensor (T/H sensor) was integrated in the sensor chamber. For this purpose, the “HYT 221—Digital Humidity and Temperature Module” was used. A display was also installed in order to observe the pollutant concentration, relative humidity and air temperature during the experiments. The electronics were operated using microcontrollers (µC). A small pump with a flow of 1 L/min was also used in order to perform active air measurements.

### 2.2. Laboratory and Field Experiments

The complete laboratory experimental plan is shown in Table 3. In total, 96 experiments were carried out under laboratory conditions. Six different levels of relative humidity and four levels of temperature were tested. In the case of the low-cost dryer implementation, four levels of relative humidity were investigated. In order to check the repeatability of the experiment, each relative humidity and temperature experiment was repeated. In the case of relative humidity, the lowest level was 10%, and it was increased in steps of 15% until 85%. Moreover, the temperature levels investigated in the laboratory were 10 °C, 25 °C, 35 °C, and 45 °C.

**Figure 2:**
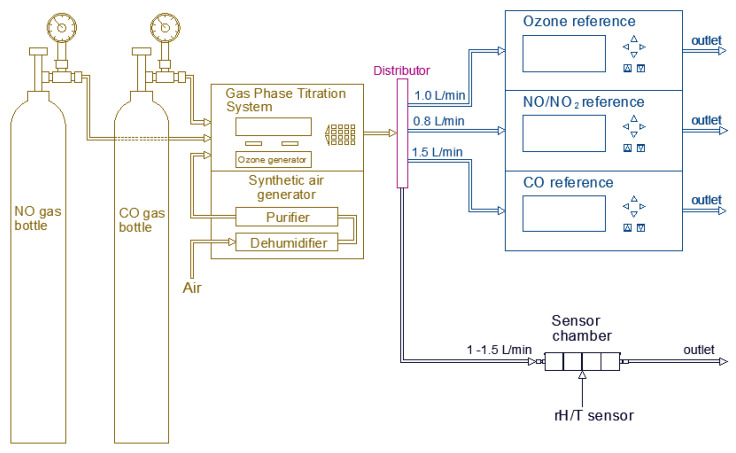
General experimental setup in the laboratory.

The general set up used during the laboratory experiments is shown in Figure 2 in which it is possible to see how the sample gas was distributed to the reference instruments and sensor chamber. An analog flow meter was used to measure the sample flow given to the low-cost sensors. The high concentrated target gas from the gas bottles was diluted using synthetic air generator. The high concentrated gas bottles for CO and NO had the initial concentrations of 101.8 ± 2.0 ppm and 121 ± 2.3 ppm, respectively. Different target gas concentrations could be achieved with the help of GPT. The NO and CO gases were diluted directly from the respective gas bottles while the O_3_ gas was produced by using an ozone generator inside the GPT. The NO_2_ gas was generated by the titration of NO and O_3_.

The target gas concentration sequences programmed in the GPT are displayed in Table 4. These concentrations were chosen by taking into account normal and extreme ambient air conditions. In preliminary tests, it was observed that the stabilization time of the low-cost sensor signal after changing a concentration step was around two minutes. Thus, each concentration step was planned with a duration of 13 min from which the first three minutes were discarded in the final analysis in order to make sure that the values were stable.

It is important to mention that the low-cost sensors measure the pollutant concentration in millivolts, which needed to be converted into the pollutant concentration in parts per billion (ppb) by applying the following equation:

(1) $$c\left(ppb\right)=\frac{\left(W_{E}-W_{E}{}_{0}\right)-\left(A_{E}-A_{E}{}_{0}\right)}{S}$$

where, *W_E_* is the value obtained from the working electrode of the low-cost sensor in millivolts; *W_E0_* is the value of the working electrode zero voltage in millivolts; *A_E_* is the value obtained from the auxiliary electrode of the low-cost sensor in millivolts; *A_E0_* is the value of the auxiliary electrode zero voltage in millivolts; *S* is the sensitivity in millivolts per ppb.

#### 2.2.1. Relative Humidity

**Figure 3:**
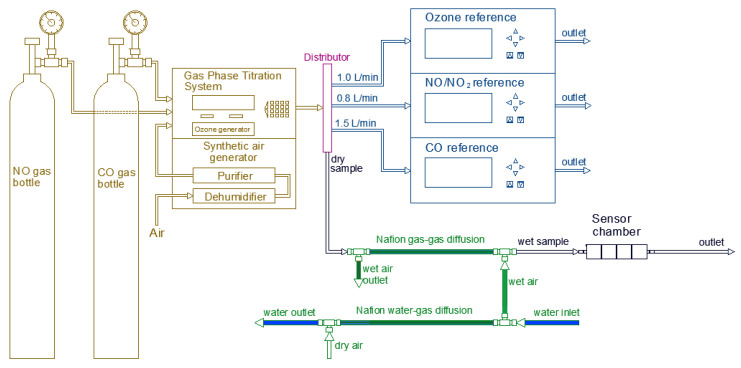
Experimental set up for testing the influence of relative humidity on low-cost gas sensors.

In order to perform the relative humidity experiments, it was required to vary the humidity in a controlled manner. To do so, a humidification system consisting of two Teflon tubes equipped with Nafion membranes (MH-110-48F-4 and MD-110-144F-4) from the company Perma Pure, a synthetic air bottle as auxiliary gas, and a Refrigerated Bath Circulator (RBC) was mounted along with the general set up as shown in Figure 3. The Nafion membranes are made of a moisture exchange material, which permits water through vapor diffusion that in turn causes the humidification or drying of a sample. In this process, the water molecules diffuse via interconnected ionic channels inside the membrane, being absorbed by sulfonic acid groups exposed to one side and pervaporates off to the opposite side of the membrane. The driving force of the reaction is the difference of H_2_O concentration (liquid or vapor) of both sides of the tubing wall. Consequently, there is no need to add any further external energy for the reaction to take place. This technology was selected due to its high temperature and pressure tolerance as well as its resistance to chemical attack and negligible reactivity against target gases [33]. It was observed that the relative humidity level achieved in the membrane depended on the temperature of the water flowing through the tube, which was controlled by RBC. In order to avoid condensation in the tube with the sample gas due to high relative humidity levels, the first tube equipped with Nafion membrane was connected directly to the RBC to get a water–gas controlled humidification of the dry air coming from the synthetic air bottle. Then the second tube containing the Nafion membrane was attached to allow a gas–gas moisture exchange of the humidified synthetic air with the sample flow that carried the sample gas containing the target concentration coming from the GPT. The humidification level of the sample was regulated by varying the temperature of the water in the RBC, which was pumped to the Nafion water–gas tube.

During each experiment, the percentage of relative humidity was kept constant as well as the temperature. The low-cost sensors and the reference instruments were exposed to the gas concentration sequence under controlled conditions of temperature and relative humidity. The same process was carried out varying the temperature of the RBC to reach six relative humidity levels. In order to evaluate specifically the influence of the humidity, it is important to avoid the variation caused by the effect of temperature. Therefore, during these experiments, the temperature level of the sample flow as well as the sensor chamber was kept stable at 25 ± 2 °C. The reference instruments were tested before the experiments against any variation in measured concentration because of relative humidity change. It was found out that the reference instruments were not affected from the variation of humidity. Therefore, the humidification system was installed only for the sample air going to the low-cost gas sensors.

#### 2.2.2. Temperature

**Figure 4:**
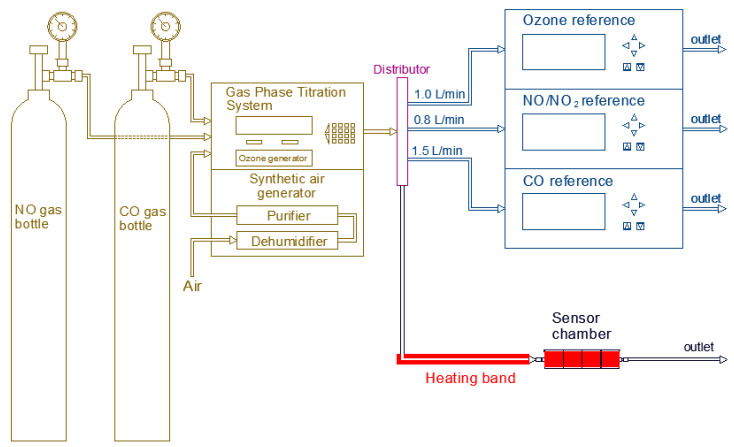
Experimental set up for testing the influence of temperature on low-cost gas sensors.

In the case of temperature, a heating band was used to cover the sensor chamber and the end of the sample tube entering the sensor chamber, as it can be observed in Figure 4. This is done in order to control the sample temperature as well as the temperature inside the sensor chamber. Similar to the relative humidity experiments, the temperature was kept stable during the whole test while the target gas concentration was applied. The behavior of the low-cost sensor was analyzed under the four different temperature levels with relative humidity level varying between 10 and 40%. This change in relative humidity was corrected using the correction algorithm calculated from the humidity experiments before. The reference instruments were also tested before the experiments using the target gas concentration at different temperature levels. The reference instruments were not affected from the temperature variation. Therefore, in this case, the variation of temperature was applied only for the sample inlet and the sensor chamber. 

#### 2.2.3. Low-Cost Dryer Implementation

**Figure 5:**
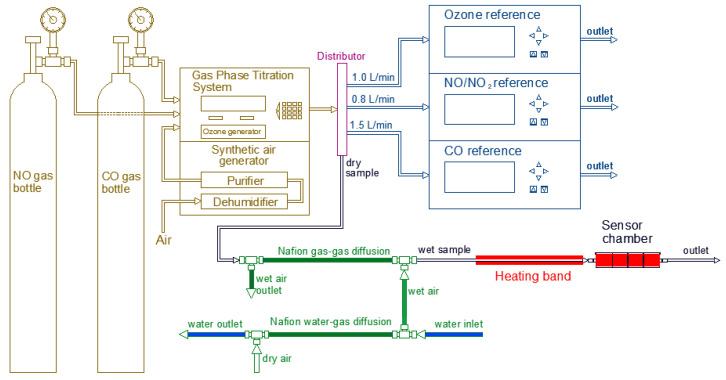
Experimental set up for testing the influence of implementing the low-cost dryer.

For the low-cost dryer implementation assessment, the two setups described in previous sections were combined. The combined setup can be seen in Figure 5. At first, the desired relative humidity level was set using the humidification system. The low-cost sensors were tested only under the four highest relative humidity levels (40%, 55%, 70%, and 85%). Moreover, 10% and 25% were not tested since they are already semi-dry conditions. Then a heating band was used as a dryer to reduce the level of relative humidity and maintaining a stable temperature in the sample flow as well as in the sensor chamber. When the temperature and relative humidity levels stabilized, the target gas concentration was given and the results were obtained. The aim was to check if the low-cost dryer was able to decrease the humidity and its effects on the low-cost gas sensors at different humidity levels.

#### 2.2.4. Field Measurements

Field measurements were carried out during the month of November 2019 in order to apply the correction algorithm developed during the laboratory experiments to the data obtained by the low-cost gas sensors on the field and compare it with the conventional devices. The low-cost gas sensor platform was installed at a monitoring station equipped with conventional measurement devices located at Marienplatz, Stuttgart. This location was selected to have a variability in the measured concentrations as the measurement location is close to the city center and has a heavy traffic road passing by.

#### 2.2.5. Performance Assessment of the Low-Cost Sensors

The performance of the correction algorithm was assessed using the following parameters.

Mean Absolute Percentage Error (MAPE):

(2) $$MAPE=\frac{\sum_{i=1}^{n}\frac{\left|X_{ref}-x_{i}\right|}{X_{ref}}}{n}$$

where, *X_ref_* is the concentration measured by the reference instrument, *x_i_* is the value obtained from the low-cost gas sensor, and *n* is the number of values to be evaluated.

The value of MAPE expresses the average of the error in percentage, when comparing the value measured by the low-cost gas sensors against the reference instrument. The ideal value without any error is 0%.

Mean Absolute Error (MAE):

(3) $$MAE=\frac{\sum_{i=1}^{n}\left|X_{ref}-x_{i}\right|}{n}$$

Similar to the MAPE, the MAE represents the error average when analyzing the low-cost gas sensors beside the reference instrument. In this case, the final value is in ppb and its ideal value is 0 ppb [34].

Linearity: it is the relation between the concentration value obtained from the sensor and the real concentration measured by the reference instrument. It indicates the slope obtained when the measured values obtained from the reference instruments are presented graphically on the *x*-axis, while the low-cost gas sensor values on the *y*-axis. The linearity is expressed in ppb_low-cost sensor_/ppb_reference_ and its ideal value is 1.0 [35].

Offset: it is the concentration value measured when no target gas is given to the low-cost gas sensor. The offset is expressed in ppb.

## 3. Results and Discussion

In this section, the results obtained from the relative humidity, temperature, low-cost dryer implementation, and field experiments as well as the procedure used for calculating the correction algorithm are discussed.

### 3.1. Laboratory Experiments

#### 3.1.1. Relative Humidity

The first part of the correction algorithm was developed by finding the influence relative humidity on the low-cost gas sensors. Here, the results obtained from each individual sensor are explained and summarized. These experiments were carried out using the relative humidity setup described in the previous section.

**Figure 6:**
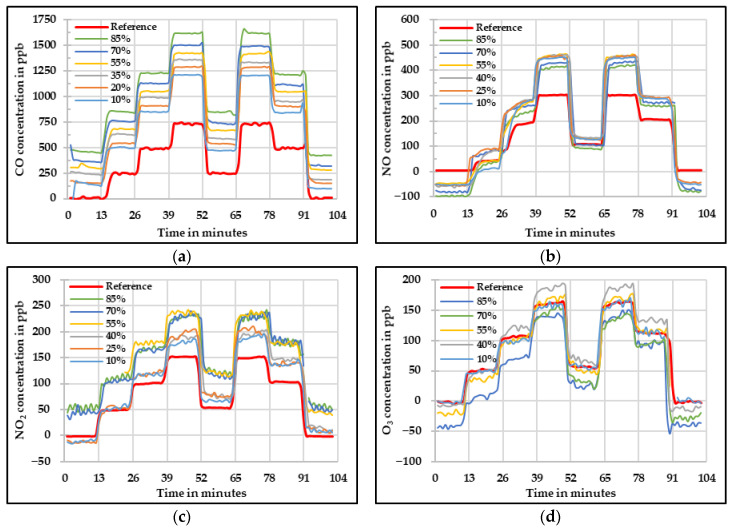
Concentration at different relative humidity levels measured by the four low-cost sensors (**a**) CO, (**b**) NO, (**c**) NO_2_, and (**d**) O_3_—raw values.

Figure 6 shows the raw values measured by the low-cost gas sensors compared against the values obtained by the reference instruments. In these figures, it is possible to see that the raw values need to be corrected because they are highly influenced by the relative humidity level. In the case of CO, a large overestimation was observed. This overestimation increased with the increase in relative humidity. In a similar way, the NO low-cost sensor overestimated the concentrations, but the increment according to the relative humidity level was not as high as it was for CO low-cost sensor. Nevertheless, a large negative offset needed to be corrected. The NO_2_ low-cost sensor showed relatively good results at low relative humidity levels. However, with the increase in relative humidity, the differences compared with the reference also increased. Additionally, it is noticeable that the signals from this NO_2_ low-cost sensor started to oscillate. This oscillation was significant at high relative humidity levels (rH ≥ 55). The O_3_ low-cost sensor showed a different behavior as the concentration tend to increase with increase in relative humidity until 40% relative humidity level. After that, the concentration started to decrease with increase in relative humidity level.

As a first step for the correction algorithm, a linear fit was calculated considering the raw values measured during the experiment carried out at 10% relative humidity and the values measured by the respective reference instruments, which was named as “baseline correction”. This 10% relative humidity baseline correction was then applied to the values measured for other five relative humidity levels in order to bring them to the same level. After bringing all the relative humidity results to the same level, the influence of change in relative humidity can be evaluated.

**Figure 7:**
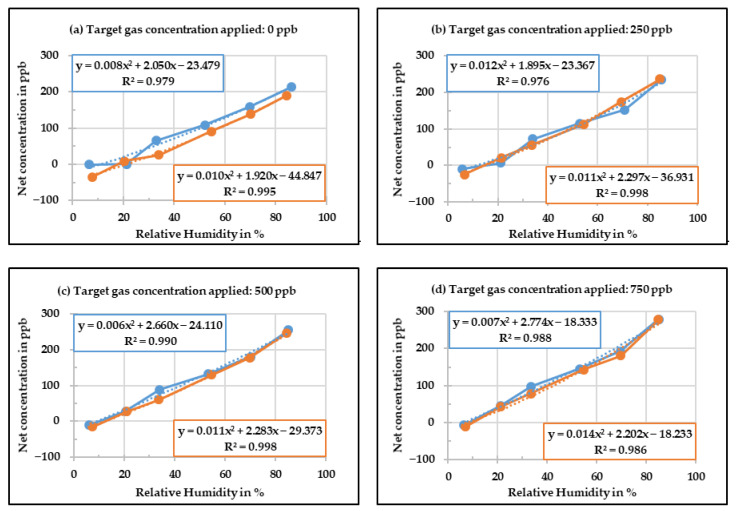
Corrected concentration measured by the CO low-cost sensor as a function of different relative humidity levels.

The next step was to develop the correction equation for relative humidity. In order to do so, the net change in concentration of the low-cost gas sensors was calculated by subtracting the concentration measured by the reference instrument from the concentration measured by the CO low-cost sensor with baseline correction. This net change in concentration was plotted against the relative humidity for different target gas concentrations and a polynomial line equation was generated. The target gas concentrations of 0 ppb, 250 ppb, 500 ppb, and 750 ppb were applied in the case of CO low-cost sensor. The diagrams presented in Figure 7 display the correlation between the concentration measured by the CO low-cost sensor with the baseline correction and the relative humidity level. For each case, the target gas concentration was applied twice, which is shown as blue and orange lines.

It can be observed in Figure 7 that with increasing relative humidity levels, the difference between the corrected value and the reference instrument increases. Although one can deduce that in the case of CO low-cost sensor, the relative humidity influence is almost linear, a polynomial equation was chosen as it had a better fit. The whole procedure was repeated twice, which in the end produced 16 polynomial equations for CO low-cost sensor. An average of these polynomial equations was obtained in order to develop the final relative humidity correction equation. 

This procedure was carried out for the other three low-cost sensors as well, which yielded the relative humidity correction equations for respective sensors:

(4) $$CO\left(rH\right)=\frac{CO_{raw}}{1.4655}+0.0074\;rH^{2}\;\left(\%\right)-3.3747\;rH\;-58.41$$

(5) $$NO\left(rH\right)=\frac{NO_{raw}}{1.685}+0.0086\;rH^{2}\;\left(\%\right)-0.5568\;rH\;+31.95$$

(6) $$NO_{2}\left(rH\right)=\frac{NO_{2}{}_{raw}}{1.234}+0.0003\;rH^{3}\;\left(\%\right)-0.0362\;rH^{2}\left(\%\right)+0.7086\;rH\;-7.57$$

(7) $$O_{3}\left(rH\right)=\frac{O_{3}{}_{raw}}{1.1318}+0.01216\;rH^{2}\;\left(\%\right)-0.6693\;rH+29.55$$

**Figure 8:**
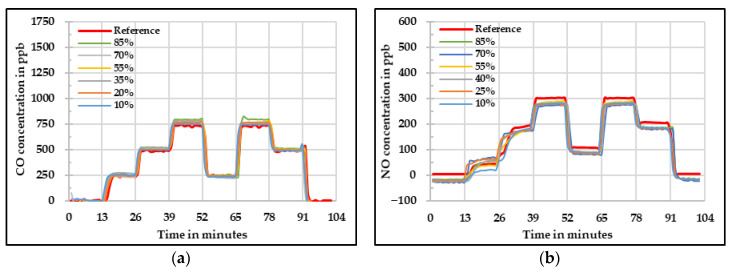

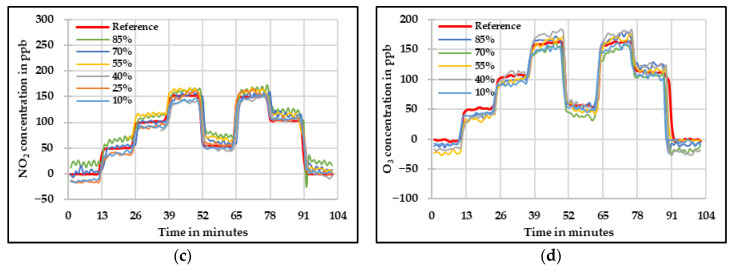
Concentration at different relative humidity levels measured by the four low-cost sensors (**a**) CO, (**b**) NO, (**c**) NO*_2_*, and (**d**) O*_3_*—correction using relative humidity developed formula.

The results of NO_2_ low-cost sensors were improved by using the correction equation until rH^3^ while for the other low-cost gas sensors no significant improvement was observed with rH^3^. Therefore, only for NO_2_ low-cost sensor the correction equation until rH^3^ was used. The results obtained after applying the Equation (4) to Equation (7) to the raw values of the low-cost gas sensors are presented graphically in Figure 8. Figure 8a presents the results of the CO low-cost sensor after applying the developed equation, it is possible to observe a clear improvement compared to the values displayed in Figure 6a. Moreover, all the evaluated parameters were greatly improved and the errors are minimized. The corrected NO concentration is shown in Figure 8b in a similar way. It can be seen that after applying the correction on the raw data, the data quality of the NO low-cost sensor was enhanced. The graphical results of NO_2_ concentrations after applying the correction algorithm are shown in the Figure 8c. In this case, one can see that although the data quality was enhanced, the signal fluctuations at extreme conditions are still noticeable. Regarding the O_3_ low-cost sensor also, the experimental results illustrate good quality after correction. Therefore, it is possible to say that the method applied for the correction of the relative humidity influence was efficient.

In general, according to the evaluated parameters, it is possible to say that the quality and reliability of the data obtained from the low-cost gas sensor were improved significantly using the relative humidity correction. Nevertheless, there was still the necessity to evaluate the temperature influence and develop a complete algorithm to correct it as well.

#### 3.1.2. Temperature

As a second part of the correction algorithm, the temperature influence was determined. The results obtained during the determination of the temperature influence on each low-cost gas sensor are discussed in this section.

**Figure 9:**
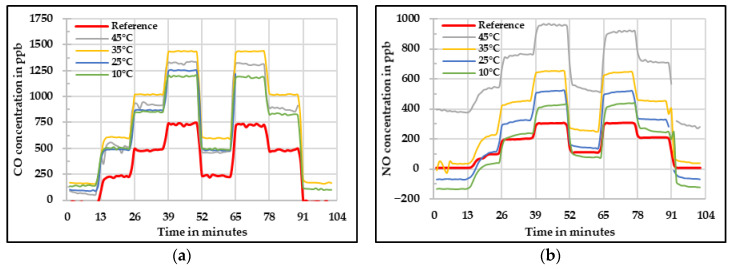

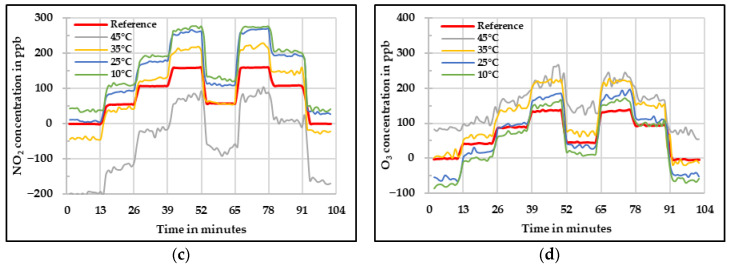
Concentration at different temperature levels measured by the four low-cost sensors (**a**) CO, (**b**) NO, (**c**) NO*_2_*, and (**d**) O*_3_*—raw values.

The raw values measured by the low-cost gas sensors at different temperature levels are shown in Figure 9. It can be seen that in the four cases, there is an influence of the different temperature levels on the concentrations obtained. In the case of the CO low-cost sensor (Figure 9a), the lowest concentrations were measured at 10 °C and the highest at 35 °C. It is interesting to notice that the values measured at 45 °C were lower compared to the ones at 35 °C. Therefore, from this analysis it is possible to conclude that the CO low-cost sensor does not have a linear behavior with respect to temperature. The raw values measured by the NO low-cost sensor are shown in Figure 9b. It is visible in this figure that the measured value increases with increasing temperature. In addition, the zero values measured at low temperatures were lower than the reference instrument but at high concentrations, the measured values were higher than the reference.

From the NO_2_ low-cost sensor results, one can infer that the influence of the temperature is not extreme at low temperatures but it is strongly pronounced under high temperature conditions with values reaching even in the range of −200 ppb during the zero concentration step. Another noticeable point in this case is that the increase in temperature is inversely proportional to the concentration measured by the NO_2_ low-cost sensor. In addition to that, at elevated temperature (45 °C) the sensor signal started to oscillate.

For the O_3_ low-cost sensor (Figure 9d), one sees that at extreme temperature conditions, the low-cost O_3_ sensor behaves poorly with unsteady signal and slow reaction time against concentration variation. In addition, similarly to what was observed for the CO and NO_2_ low-cost sensors, the difference between the concentrations measured at 10 °C and 25 °C is small. Nevertheless, a greater difference is observed when comparing the values at 10 °C and 45 °C. Therefore, from this analysis, one can conclude that a suitable range of temperatures for the low-cost sensors to work avoiding a large temperature influence would be between 10 °C and 25 °C.

**Figure 10:**
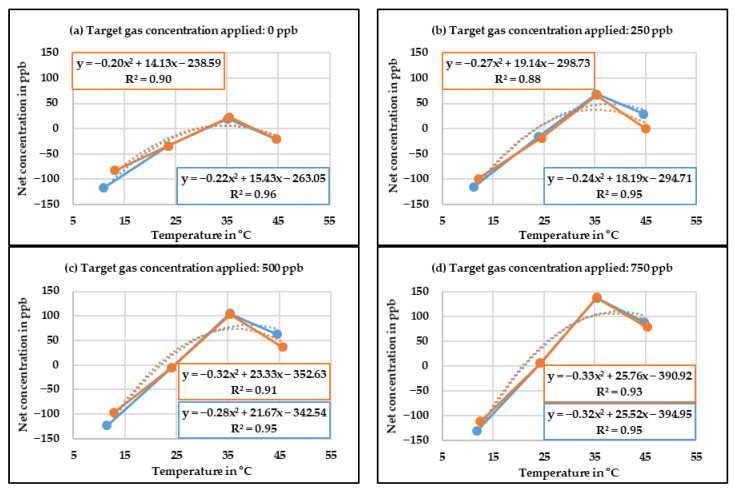
Corrected concentration measured by the CO low-cost sensor as a function of different temperature levels.

During the investigation of the temperature influence, the relative humidity level in the sensor chamber varied between 10% and 40%. This variation was compensated by applying the relative humidity correction equations developed in the previous section for each respective low-cost gas sensor. Since the temperature used during the relative humidity influence experiments was around 25 °C, the closest fit to the reference instrument during this set of experiments was observed for the temperature level of 25 °C. In a similar way as it was done for the relative humidity influence correction, the correlation between the corrected concentration measured by the low-cost gas sensors and the temperature level was calculated along with the necessary components for the correction equation. This analysis is shown in Figure 10 for the CO low-cost sensor. The same target gas was applied twice, which is shown as blue and orange lines in the figure. Same procedure was repeated for the other three low-cost gas sensors. The drop in concentration at higher temperatures was not expected and, therefore, very interesting. It is assumed that the higher temperature affects the reaction inside the electrochemical sensor. Further investigation is required to examine the low-cost gas sensor behavior at higher temperatures.

After doing this evaluation, the next equations were developed combining the two influencing parameters (relative humidity and temperature):

(8) $$CO\left(rH,\;T\right)=\frac{CO_{raw}}{1.4655}+0.0074\;rH^{2}\;\left(\%\right)-3.3747\;rH+0.2692\;T^{2}\left({}^{\circ}C\right)-20.3221\;T\left({}^{\circ}C\right)+265.90$$

(9) $$NO\left(rH,\;T\right)=\frac{NO_{raw}}{1.685}+0.0086\;rH^{2}\;\left(\%\right)-0.5568\;rH-0.2258\;T^{2}\left({}^{\circ}C\right)+6.0402\;T\left({}^{\circ}C\right)+18.63$$

(10) $$NO_{2}\left(rH,\;T\right)=\frac{NO_{2}{}_{raw}}{1.234}+0.0003\;rH^{3}\;\left(\%\right)-0.0362\;rH^{2}\left(\%\right)+0.7086\;rH+0.1947\;T^{2}\left({}^{\circ}C\right)-7.0152\;T\left({}^{\circ}C\right)+36.23$$

(11) $$O_{3}\left(rH,T\right)=\frac{O_{3}{}_{raw}}{1.1318}+0.01216\;rH^{2}\left(\%\right)-0.6693\;rH-0.3133\;T^{2}\;\left({}^{\circ}C\right)+18.9387\;T\;\left({}^{\circ}C\right)-285.28$$

**Figure 11:**
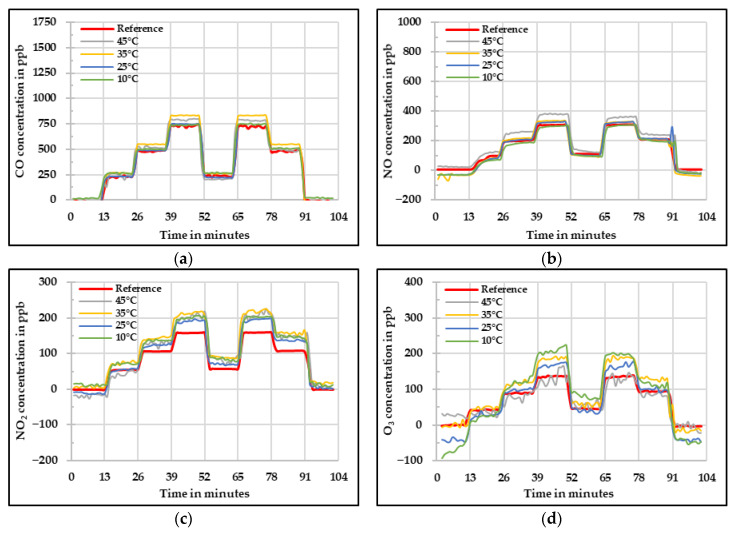
Concentration at different temperature levels measured by the four low-cost sensors (**a**) CO, (**b**) NO, (**c**) NO*_2_*, and (**d**) O*_3_*—correction using combined developed formula.

The graphical results obtained by applying the above equations on the raw data acquired from the low-cost gas sensors are presented in Figure 11. In this figure, it is evident that the influence of the change in temperature was reduced. However, there is still a small offset for the high concentrations during the time in which the temperature was 35 °C for CO and 45 °C for NO. In the case of NO_2_, the quality of the values at low concentration were highly improved for all of the temperatures. However, the values at high concentrations were influenced by the extremely low signal measured at 45 °C. Finally, the O_3_ low-cost sensor showed the most unfavorable behavior with offsets at high concentrations.

The parameters defined for the performance assessment of the relative humidity correction are summarized in Table 5. The Table 6 summarizes the assessment carried out for the developed temperature influence correction. It is observable that the method applied to correct the humidity and temperature correction worked in a quite satisfactory manner since the errors as well as the offset were drastically reduced and the linearity was brought to a value near 1.0. The optimum values were obtained for the CO and NO measurements, with linearity and MAPE between 1.0–1.1 and 0.2–1.2, respectively. Nevertheless, as it was expected by analyzing the graphical results of the NO_2_ and O_3_, the results were improved but not satisfactory as the ones observed for the previous low-cost sensors. Especially at high temperatures, these two low-cost sensors behaved poorly and further effort would be needed to get more reliable measurements. 

#### 3.1.3. Low-Cost Dryer Implementation

**Figure 12:**
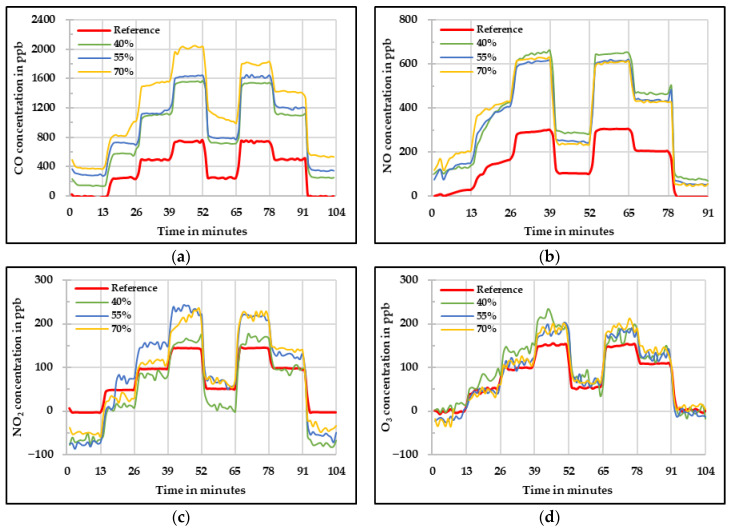
Concentration at different relative humidity levels measured by the four low-cost sensors (**a**) CO, (**b**) NO, (**c**) NO*_2_*, and (**d**) O*_3_* using low-cost dryer—raw values.

It is known that the values measured by using the low-cost gas sensors are influenced by change in relative humidity and temperature. One method to compensate this influence is to quantify the effect of these parameters and apply the correction to the raw values. Another method is to keep these parameters constant in order to avoid any influence. This can be done by using a low-cost dryer that can keep the sample air temperature and relative humidity constant. This method was also tested in this research. The results obtained for the low-cost dryer implementation assessment are shown below. Figure 12 shows the raw concentrations measured by the four low-cost gas sensors at different relative humidity levels. It is important to mention that the humidity levels mentioned here are the values set in the sample flow before the drying process. During the whole set of experiments, the selected temperature for the low-cost dyer was between 40 °C and 42 °C and the relative humidity levels varied in a narrow range between 18% and 30%. The main idea of using the low-cost dryer was to check if the impact of these two influencing parameters on the low-cost gas sensors can be reduced.

Figure 12a shows the raw values measured by the CO low-cost sensor. In this figure, one can see that the measured concentration overestimates the real concentration obtained by the reference instrument in all three cases. At 40% and 55% relative humidity, the values are near to each other. Additionally, the values obtained during the experiment at 70% relative humidity for the CO low-cost sensor slightly differ from the starting phase to the ending phase. For the NO low-cost sensor (Figure 12b), it is visible that there is not a great difference between the different relative humidity levels and the concentration difference compared to the reference device is mainly due the offset. Thus, one can say that there is not any perceivable influence of the different relative humidity levels.

The raw values measured by the NO_2_ low-cost sensor are presented in Figure 12c. It is possible to see that there is an underestimation when zero and low concentrations are given. On the other hand, there is an overestimation at high concentrations. Besides a similar behavior as the one observed during the temperature experiments at high temperatures, the sensor signal oscillated that affected afterwards the results of the MAE and MAPE. Lastly, signal fluctuation was observed by measuring O_3_. This might be due to the high temperature at which the low-cost O_3_ sensor was exposed causing this wavering in the sensor signal. Moreover, regarding the quality of the data, there is an overestimation at high concentrations, but it is important to see that the sensor had a quite homogeneous behavior under the three different relative humidity levels.

**Figure 13:**
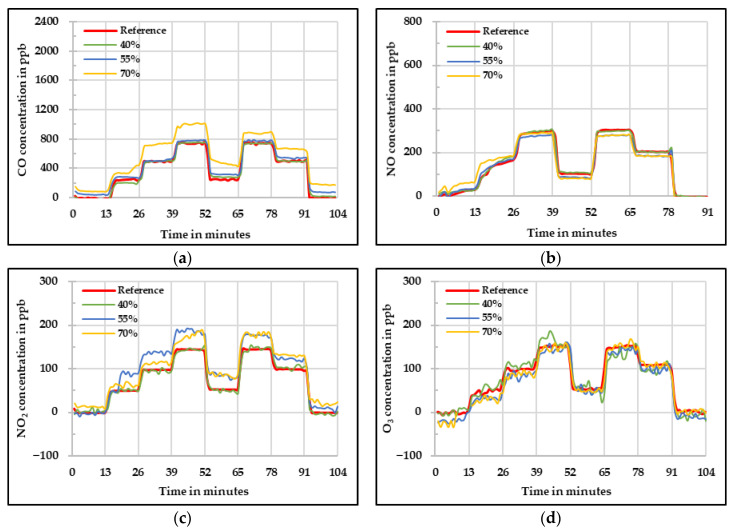
Concentration at different relative humidity levels measured by the four low-cost sensors (**a**) CO, (**b**) NO, (**c**) NO_2_, and (**d**) O_3_ using low-cost dryer—corrected values.

A linear fit was applied to all the measured values taking into account the results at 40% relative humidity and reference instrument. These results are shown in Figure 13 for each respective low-cost gas sensor. For CO, it can be seen in Figure 12a) that according to what was expected, the influence of the relative humidity was drastically reduced by the use of the low-cost dryer. For 40% and 55% relative humidity, the results are satisfactory. However, for 70% relative humidity, the starting phase shows higher deviation leading to higher measurement uncertainty in this phase. The greatest enhancement was observed for the NO, in which no observable difference between the three relative humidity levels was achieved. In this case, the implementation of the low-cost dryer was suitable.

The influence of the relative humidity at low concentrations was not so significant for the NO_2_ low-cost sensor. However, at high concentrations the sensor was still affected even with the usage of the low-cost dryer. For the low-cost O_3_ sensor, it is possible to see that the values at the three different relative humidity levels were improved and brought to the reference measured concentration. However, the fluctuation in the signal was observed.

In Table 7 the statistical assessment of the low-cost dryer implementation on the four low-cost sensors is shown. It is visible that by using the low-cost dryer, the effort to develop a correction algorithm is greatly reduced. This gives an idea that such a solution can also help to improve the data quality obtained from the low-cost gas sensors. The development of a low-cost dryer can be optimized according to the measurement setup being used. Nevertheless, the results are not as reliable as the ones obtained by calculating the correction algorithm based on the relative humidity and temperature influences on the low-cost gas sensors. The implementation of a low-cost dryer could be considered as a solution to have an idea of the pollutant behavior in a determined zone. Further correction would be needed in the case of CO and NO_2_. Additionally, the signal fluctuation behavior can be reduced by applying long-term averages. Furthermore, there can be the possibility that different low-cost gas sensors require different correction coefficients for relative humidity and temperature correction equation. Then the necessity of carrying out the same experiments to determine them would be avoided by the implementation of this technology.

### 3.2. Field Experiments

**Figure 14:**
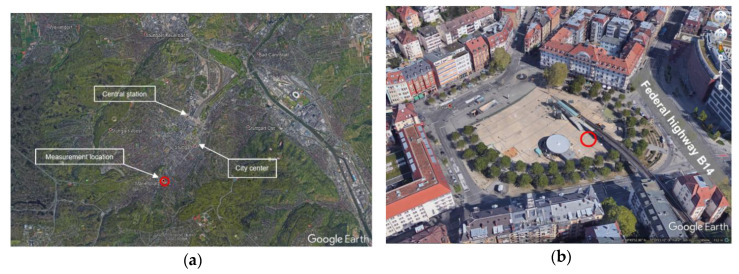
Location of reference monitoring station used for field measurements indicated with red circle (**a**) aerial view of Stuttgart (**b**) aerial view of Marienplatz.

The purpose of field measurements was to analyze the performance of the low-cost gas sensors under ambient conditions. The measurements were done at a stationary monitoring station located at Marienplatz close to the city center of Stuttgart (48°45′50.7′′ N 9°10′07.4′′ E). The measurement location is shown with the red circle as it can be seen in Figure 14a,b. Figure 14a shows the aerial view of Stuttgart and Figure 14b shows the aerial view of Marienplatz. This location is a hotspot for air pollution near the city center of Stuttgart. The main pollution source near this station is traffic, as a federal highway with an average traffic volume of more than 50,000 vehicles per day is nearby [36].

The low-cost gas sensors were installed for one month (November 2019) with the professional devices for further comparisons. The correction algorithms developed in the laboratory from the results of the relative humidity and temperature experiments were applied to the field data in order to correct and increase the quality of the data obtained by the low-cost gas sensors. The corrected results were then compared with the professional devices. Since the measurement was a long-term observation, it was decided to take hourly averages instead of minutely average as it was done during the laboratory experiments. This avoided a big data set and provided better results. 

**Figure 15:**
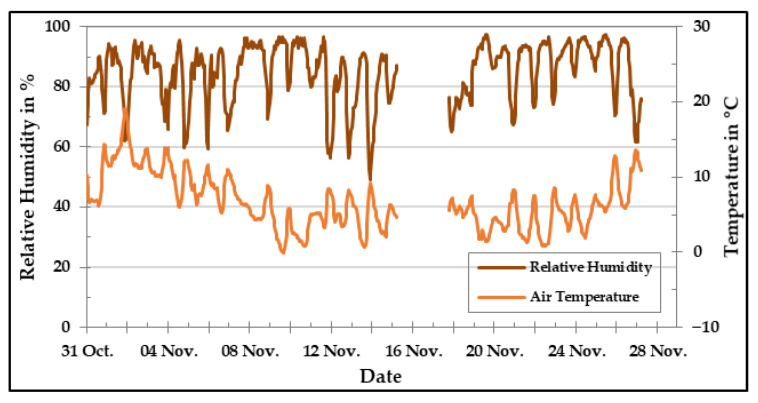
Relative humidity and temperature level in the field during November 2019.

Figure 15 presents the relative humidity and temperature levels measured during the field experiments. As it can be seen, the relative humidity levels were high during the whole measurement, between 50% and 94%. In addition to that, the temperature was in the range from 0 °C to 18 °C. The day-night cycles can be clearly observed in both cases.

**Figure 16:**
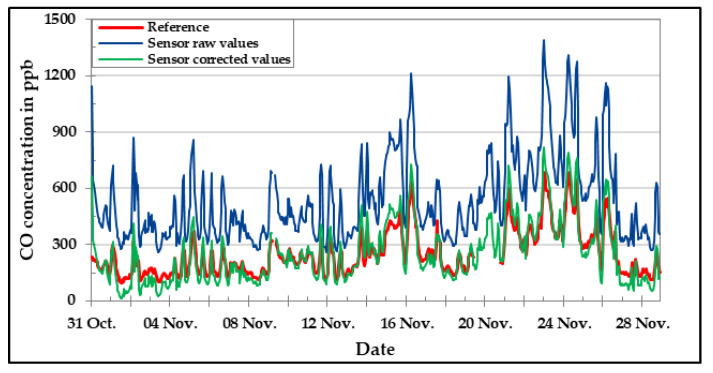
CO concentration in the field during November 2019—comparison between reference instrument, raw, and corrected sensor data.

**Figure 17:**
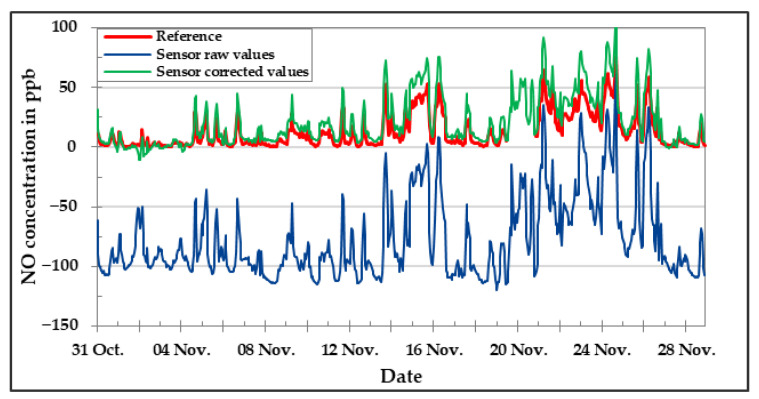
NO concentration in the field during November 2019—comparison between reference instrument, raw, and corrected sensor data.

**Figure 18:**
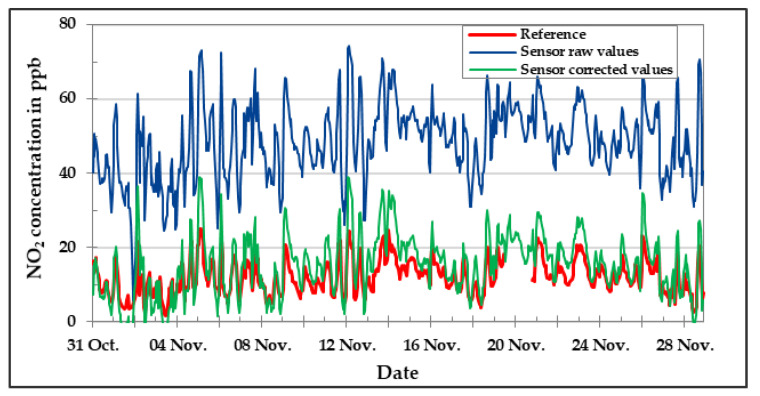
NO_2_ concentration in the field during November 2019—comparison between reference instrument, raw, and corrected sensor data.

Figure 16, Figure 17 and Figure 18 show the results of CO, NO, and NO_2_ low-cost sensors, respectively, during the field experiment. Unfortunately, the O_3_ low-cost sensor could not be included in the field analysis due to technical problems. The comparison between the concentration values of the target pollutant measured by the reference instruments and the corresponding low-cost gas sensor results are presented for the raw values as well as the values obtained after applying the correction algorithm developed in the laboratory. One can clearly see by looking at the graphics that the correction equations worked in a satisfactory manner. The deviation between sensor data and reference data could be reduced drastically by applying correction algorithms developed in the laboratory. Anyhow, there are still some deviations to be seen.

The statistical analysis using the defined parameters are given in Table 8. From that table, it is evident that the improvement of the error as well as the offset is considerable in all the cases. However, the values for corrected linearity were not satisfactory for NO and NO_2_ low-cost sensor. This might be because of the concentration ranges, which were below the lowest points analyzed during the laboratory experiments. Moreover, the results obtained from the offset of the CO low-cost sensor are too large compared to the other low-cost sensors.

## 4. Conclusions

This study proved that the low-cost gas sensors could provide a great opportunity to obtain good spatial distribution data of air pollutants or ambient air quality. It is possible to measure air quality using low-cost electrochemical gas sensors after applying quality assurance measures, i.e., processing the raw data and correcting the effects of the factors that influence the functionality of these low-cost gas sensors. With the obtained results, it is possible to get an accuracy of above 70% and in some cases above 90% if appropriate post-processing of the data is performed. The laboratory experiments helped to understand the behavior of the low-cost gas sensor signal when the relative humidity or the air temperature was varying in a wide range. Additionally, it was possible to quantify the increase or decrease of concentration in ppb caused by the changes in relative humidity or air temperature on the low-cost gas sensors and develop correction algorithm to reduce these influences, even under extreme conditions. In most of the cases, the applied algorithm was suitable to improve the data quality. The CO, NO, and NO_2_ low-cost sensors showed satisfactory improvement after applying the correction algorithm, while the O_3_ low-cost sensor showed the least improvement. Some of the low-cost gas sensors observed fluctuations under high temperature conditions (>35 °C), which should be examined intensively.

Implementing a low-cost dryer, which maintains the temperature and relative humidity in the sensor chamber constant, decreases the effort of developing a complex correction algorithm. Therefore, in the case that each low-cost gas sensor has different relative humidity and temperature influences, a simple linear regression model can increase the data quality. The disadvantage is to implement the low-cost dryer system to the measurement platform and constant energy supply.

The field experiment demonstrated that the assessment carried out in laboratory could simulate the outdoor conditions in a correct manner and correct them in a way that the influences of such conditions do not affect the reliability of the low-cost gas sensors data. The analyzed errors (MAPE and MAE) were significantly reduced and the linearity was suitable within the measured concentrations.

As a final conclusion, the results of this investigation showed that the low-cost gas sensors can be used as a good support for the current traditional air quality monitoring stations. This will permit the feasibility of the creation of a wide and detailed air quality map, which would give precise information for the development and application of appropriate actions to tackle the air pollution.

## Figures and Tables

**Table 1:** Electrochemical low-cost sensors used in this research.

Sensor	Physical Characteristics	Range of Operation
CO-B4/CO gas	Weight: 13 g each Dimensions: cylinder with 32.3 mm diameter and 16.5 mm height	Temperature: −30 °C to 50 °C Relative Humidity: 15% to 90%
NO-B4/NO gas		
NO2-B43F/NO_2_ gas		
OX-B431/O_3_ gas		

**Table 2:** Reference devices used in laboratory and field experiments.

Gas	Device	Principle	Time Resolution (s)	Accuracy
CO	CO Monitor—Model APMA-360 Horiba Company	Non-dispersive infrared absorptiometry (NDIR)	1	±1%
NO, NO_2_	NO_X_ Monitor—Model 200A MLU Company	Chemiluminescence	1	±0.5%
O_3_	Ozone Monitor—Model APOA-360 Horiba Company	Non-dispersive ultraviolet-absorption (NDUV)	1	±2%

**Table 3:** Laboratory experimental plan.

Parameter	Low-Cost Sensors	Levels	Repetition	Number of Experiments
Relative Humidity	4	6	2	48
Temperature	4	4	2	32
Low-cost dryer	4	4	1	16

**Table 4:** Target gas concentration sequences programmed in Gas Phase Titration (GPT) for the experimental plan.

Target Gas	Concentrations (ppb)							
CO	0	250	500	750	250	750	500	0
NO	0	100	200	300	100	300	200	0
NO_2_	0	50	100	150	50	150	100	0
O_3_	0	50	100	150	50	150	100	0

**Table 5:** Relative humidity correction assessment using Mean Absolute Percentage Error (MAPE), Mean Absolute Error (MAE), linearity, and offset.

rH Level		10%				25%				40%				55%				70%				85%			
Parameter		CO	NO	NO_2_	O_3_	CO	NO	NO_2_	O_3_	CO	NO	NO_2_	O_3_	CO	NO	NO_2_	O_3_	CO	NO	NO_2_	O_3_	CO	NO	NO_2_	O_3_
MAPE	Raw	2.8	2.7	1.5	2.5	7.5	2.3	1.9	15.2	12.4	2.3	1.9	2.9	9.2	1.9	5.2	2	10	3.2	6.4	3.1	10.6	3.9	6.1	6.8
-	Corrected	0.6	1.0	1.3	0.4	0.9	1.0	1.4	6.1	0.8	1.0	0.4	1.1	0.8	0.7	1	0.7	0.9	1.2	2.8	0.7	0.9	1	2.3	2.6
MAE	Raw	315.9	75.5	2.1	24.3	369.2	78.0	25.9	102.4	432.8	89.6	34.6	21.4	514.8	90.5	71.9	25.5	593	72.3	65.2	48.9	678	73.6	70.1	61.6
ppb	Corrected	22.6	20.7	7.7	6.6	24.2	20.9	8.4	79.0	40.0	20.9	6.6	13.1	25.7	16.7	13.3	10.6	27	25.3	7.2	13.3	37.7	18.6	17.8	9.3
Linearity	Raw	1.5	1.7	1.2	1.1	1.5	1.7	1.3	0.3	1.5	1.8	1.2	1.3	1.5	1.7	1.2	1.3	1.6	1.7	1.2	1.2	1.6	1.7	1.2	1.3
ppb_sensor_/ppb_reference_	Corrected	1.0	1.0	1.0	1.0	1.0	1.0	1.1	0.2	1.1	1.1	1.0	1.2	1.1	1	1	1.2	1.1	1	1	1	1.1	1	0.9	1.1
Offset	Raw	139.5	−58.7	0.0	−34.4	166.2	−56.2	−0.8	−45.0	283.6	−61.0	20.9	−42.2	309.1	−40	49.2	−59	369	−79	48.2	−66	452	−96	57.2	−82
ppb	Corrected	0.8	−19.8	−4.2	−5.2	−16.2	−22.8	−5.8	−19.0	0.9	−26.7	2.7	−16.0	−12	−11	12.1	−23	−28	−26	6.7	−18	−28	−22.7	22.7	−9.5

**Table 6:** Temperature and relative humidity correction assessment using MAPE, MAE, linearity, and offset.

Temp Level		10 °C				25 °C				35 °C				45 °C			
Parameter		CO	NO	NO_2_	O_3_	CO	NO	NO_2_	O_3_	CO	NO	NO_2_	O_3_	CO	NO	NO_2_	O_3_
MAPE	Raw	2.5	4.1	2.5	1.4	1.7	2.5	2.4	1.2	3.2	1.8	3.4	1.1	1.6	11.8	19.5	3.4
-	Corrected	0.6	1.1	0.7	1.1	0.2	0.9	0.7	1.0	0.4	1.2	1.1	0.9	0.6	0.7	1.4	0.7
MAE	Raw	320.0	83.4	83.9	26.7	307.4	111.1	64.6	26.5	453.1	192.3	32.5	40.0	408.7	483.8	128.8	69.8
ppb	Corrected	33.1	20.5	3.1	38.8	22.0	18.7	20.1	19.0	55.3	24.1	38.1	22.3	1.6	11.8	19.5	3.4
Linearity	Raw	1.4	1.8	1.5	1.6	1.5	2.0	1.6	1.6	1.6	2.0	1.6	1.5	1.6	1.8	1.7	1.0
ppb_sensor_/ppb_reference_	Corrected	1.0	1.1	1.2	1.7	1.0	1.2	1.3	1.4	1.1	1.2	1.3	1.4	1.1	1.1	1.4	0.8
Offset	Raw	164.8	−120.6	39.0	−66.4	130.9	−77.7	11.0	−47.3	221.8	25.3	−32.7	−27.4	151.5	397.0	−193.5	76.5
ppb	Corrected	46.1	−22.1	11.7	−30.2	−15.5	−31.0	−7.2	−27.4	4.3	−36.7	−13.1	−1.3	−29.0	17.1	−12.1	9.7

**Table 7:** Low-cost dryer implementation assessment using MAPE, MAE, linearity, and offset.

rH Level		40%				55%				70%			
Parameter		CO	NO	NO_2_	O_3_	CO	NO	NO_2_	O_3_	CO	NO	NO_2_	O_3_
MAPE	Raw	5.3	8.2	7.3	1.1	5.7	4.5	6.8	1.3	4.5	3.7	3.9	1.1
-	Corrected	0.7	0.4	0.4	0.7	0.9	0.8	0.7	1.2	1.2	0.7	1.4	0.7
MAE	Raw	531	219.4	33.1	25.1	628.9	193.7	51.8	21.0	913.2	202.9	40.3	25.4
ppb	Corrected	23.1	6.0	4.2	11.8	39.1	16.7	28.3	11.7	187.9	19.2	24.6	10.0
Linearity	Raw	1.8	1.9	1.6	1.2	1.7	1.9	2.0	1.3	1.9	1.8	1.7	1.4
ppb_sensor_/ppb_reference_	Corrected	1.0	1.0	1.0	1.0	1.0	1.0	1.1	1.1	1.1	1.0	1.1	1.1
Offset	Raw	215.3	88.9	−70.3	4.3	320.3	58.6	−53.6	−13.5	488.9	63.5	−41.0	−13.2
ppb	Corrected	−0.2	1.6	−0.5	−1.0	58.1	−14.6	9.9	−15.6	151.6	−12.0	17.7	−15.1

**Table 8:** Correction equation assessment using MAPE, MAE, linearity, and offset for CO, NO, and NO_2_ low-cost sensors on field data.

Field Measurement				
Parameter		CO	NO	NO_2_
MAPE	Raw	1.2	37.1	3.4
-	Corrected	0.2	1.2	0.4
MAE	Raw	281.4	91.6	36.1
ppb	Corrected	38.2	7.6	4.3
Linearity	Raw	1.8	2.2	2.0
ppb_sensor_/ppb_reference_	Corrected	1.3	1.4	1.7
Offset	Raw	77.5	107.5	23.8
ppb	Corrected	−79.5	1.4	−5.5

## Appendix A

**Table A1:** Specifications of some of the low-cost NO_2_ sensors available in the market.

Company	Alphasense (UK) ^a^	Winsen (China) ^b^	SGX (Swiss) ^c^	City Technology (UK) ^d^	SPEC Sensors ^e^	Membrapor (FR) ^f^
Model	B43F	ZE12	SGX-4NO2 - 4 (Industrial)	A3OZ EnviroceL	DGS -NO2 968-043	NO2/S-100
Sensor technology	Electrochemical	Electrochemical	Electrochemical	Electrochemical	Electrochemical	Electrochemical
Price in Euro	~200	~320	~105	~350	~	~200
Range	0–20 ppm	-	0–30 ppm	0–10 ppm	0–10 ppm	0–100 ppm
Resolution	15 ppb	<10 ppb	0.1 ppm	20 ppb (at 20 °C)	20 ppb	<0.2 ppm
Response time t_90_ in (s)	<60 from zero to 2 ppm	<30	<30	<40	<30	<60
Sensitivity	425 nA/ppm at 2 ppm	n/a	600 ± 150 nA/ppm	n/a	n/a	n/a
Linearity	<±0.5 ppm	n/a	linear	linear	n/a	linear
Dynamic range	max. 50 ppm	n/a	max. 200 ppm	max. 100 ppm	n/a	max. 600 ppm
Power requirements	ISB, 1 mA	DC 5.0 V ±0.1 V	1.3 V-, <1.0 A	n/a	n/a	n/a
Dimensions (mm)	32 × 16.5	39 × 44	20 × 20.4	42.5 × 17.8	44.5 × 20.8 × 8.9	20.4 × 41
Weight (g)	<13	75	5	22	57	32
Environmental limits	−30 to 40°C; 80 to 120 kPa and 15% to 85% RH	−20 to 50°C; 15% to 90% RH	−30 to 50°C; 80 to 120 kPa and 15% to 90% RH	−20 to 50°C; atmospheric ±10%, 15% to 90% RH	−20 to 40°C, 15% to 95% RH	−40 to 50°C, 15% to 90% RH
Lifetime	>12 months until 50% of original	2 years	2 years	2 years	>5 years	2 years in the air

^a^ Alphasense Ltd. (2019): NO2-B43F Nitrogen Dioxide Sensor 4-Electrode. Technical Specification. Alphasense Ltd. Available online at http://www.Alphasense.com/WEB1213/wp-content/uploads/2019/09/NO2-B43F.pdf, checked on 24 July 2020. ^b^ Zhengzhou Winsen Electronics Technology Co., Ltd. (2016): Electrochemical Gas Detection Module. User’s Manual V1.2—Model: ZE12. Zhengzhou Winsen Electronics Technology Co., Ltd. Available online at https://www.winsen-sensor.com/d/files/PDF/Gas%20Sensor%20Module/Industrial%20Application%20Gas%20Sensor%20Module/ze12-electrochemical-module-manualv1_5.pdf, checked on 24 July 2020. ^c^ SGX Sensortech (2020): SGX-4NO2 Datasheet. Industrial Nitrogen Dioxide (NO2) Sensor. SGX Europe Sp. z o.o., updated on 27 March 2020, checked on 24 July 2020. ^d^ City Technology Limited (2001): Ozone/Nitrogen dioxide EnviroceL^®^ Specification. With assistance of Shawcity Limited. City Technology Ltd. Available online at https://www.shawcity.co.uk/documents/SensorPDFs2017/General-air-COA3OZ-EnviroceL.pdf, updated on 10 January 2001, checked on 24 July 2020. ^e^ SPEC Sensors LLC (2017): Digital Gas Sensor—Nitrogen Dioxide. SPEC Sensors LLC. Available online at https://media.digikey.com/pdf/Data%20Sheets/SPEC%20Sensors%20PDFs/968-043_9-6-17.pdf, updated on August 2017, checked on 24 July 2020. ^f^ Membrapor AG (2020): Nitrogen Dioxide Gas Sensor NO2/S-100. Specification Sheet. Membrapor AG. Available online at http://www.membrapor.ch/sheet/Nitrogen-Dioxide-Gas-Sensor-NO2-S-100.pdf, updated on May 2020, checked on 24 July 2020.

**Table A2:** Specifications of some of the low-cost CO sensors available in the market.

Company	Alphasense (UK) ^g^	Winsen (China) ^b^	SGX (Swiss) ^h^	City Technology (UK) ^i^	Langan DataBear ^j^	Membrapor (FR) ^k^
**Model**	CO-B4	ZE12	SGX-7CO (Industrial)	A3CO EnviroceL	T 15	CO/SF-1000
**Sensor technology**	Electrochemical	Electrochemical	Electrochemical	Electrochemical	Electrochemical	Electrochemical
**Price in Euro**	~150	~320	~40	~280	~1300	~200
**Range**	0–1000 ppm	-	0–1000 ppm	0–500 ppm	0–128 ppm	0–1000 ppm
**Resolution**	4 ppb	<10 ppb	<0.5 ppm	100 ppb	0.5 ppm	<0.5 ppm
**Response time t_90_ in (s)**	<25 from zero to 10 ppm	<30	<30	<40	n/a	<50
**Sensitivity**	525 nA/ppm at 2 ppm	n/a	100±20 nA/ppm	n/a	n/a	n/a
**Linearity**	20 to 35 ppb	n/a	linear	linear	n/a	linear
**Dynamic range**	max. 2000 ppm	n/a	max. 2000 ppm	max. 1000 ppm	n/a	max. 2000 ppm
**Power requirements**	ISB, 1 mA	DC 5.0 V ±0.1 V	1.3 V; <1.0 A	n/a	n/a	n/a
**Dimensions (mm)**	32 × 16.5	39 × 44	31.5 × 15.5	42.5 × 17.8	130 × 90 × 60	20.4 × 41
**Weight (g)**	<13	75	n/a	22	575	32
**Environmental limits**	−30 to 50 °C; 80 to 120 kPa and 15% to 90% RH	−20 to 50 °C; 15% to 90% RH	−30 to 50 °C; 80 to 120 kPa and 15% to 90% RH	−20 to 50 °C; atmospheric ±10%, 15% to 90% RH	−18 to 53 °C	−40 to 50 °C, 15% to 90% RH
**Lifetime**	>24 months until 50% of original	2 years	1 year	2 years in air	n/a	3 years in air

^g^ Alphasense Ltd. (2019): CO-B4 Carbon Monoxide Sensor 4-Electrode. Technical Specification. Alphasense Ltd. Available online at http://www.alphasense.com/WEB1213/wp-content/uploads/2019/09/CO-B4.pdf, updated on July 2020, checked on 24 July 2020. ^b^ Zhengzhou Winsen Electronics Technology Co., Ltd. (2016): Electrochemical Gas Detection Module. User Manual V1.2—Model: ZE12. Zhengzhou Winsen Electronics Technology Co., Ltd. Available online at https://www.winsen-sensor.com/d/files/PDF/Gas%20Sensor%20Module/Industrial%20Application%20Gas%20Sensor%20Module/ze12-electrochemical-module-manualv1_5.pdf, checked on 24 July 2020. ^h^ SGX Sensortech (2016): SGX-7CO Datasheet. Industrial Carbon Monoxide Sensor. SGX Europe Sp. z o.o. Available online at https://www.sgxsensortech.com/content/uploads/2014/07/DS-0144-SGX-7CO-V4.pdf, updated on 29 July 2016, checked on 24 July 2020. ^i^ City Technology Limited (2018): Carbon Monoxide (CO) Gas Sensor. Product Data Sheet. City Technology Limited. Available online at https://www.citytech.com/en-gb/PDF-Datasheets/a3co.pdf, updated on 22 January 2018, checked on 24 July 2020. ^j^ Langan Products, Inc. (1997): Langan Model T15 CO. Data Summary. Langan Products, Inc. Available online at http://www.langan.biz/CO/, updated on 3 February 1997, checked on 24 July 2020. ^k^ Membrapor AG (2020): Carbon Monoxide Gas Sensor CO/SF-1000. Specification Sheet. Membrapor AG. Available online at http://www.membrapor.ch/sheet/Carbon-Monoxide-Gas-Sensor-CO-SF-1000.pdf, updated on April 2020, checked on 24 July 2020.

**Table A3:** Specifications of some of the low-cost NO sensors available in the market.

Company	Alphasense (UK) ^l^	SGX (Swiss) ^m^	City Technology (UK) ^n^	DD Scientific	Pro Sense (China) ^p^	Membrapor (FR) ^q^
Model	B4	EC 4-250-NO	NO 3E 100	GS+7NO	4NO-250	NO/S-100
Sensor technology	Electrochemical	Electrochemical	Electrochemical	Electrochemical	Electrochemical	Electrochemical
Price in Euro	~250	~103	n/a	n/a	n/a	~200
Range	0–20 ppm	0–250 ppm	0-100 ppm / 0-500 ppm	0–100 ppm	0–250 ppm	0–100 ppm
Resolution	15 ppb	0.5 ppm	<0.7 ppm (at 20 °C)	0.5 ppm	n/a	<0.5 ppm
Response time t_90_ in (s)	<45 from zero to 2 ppm	<35	<20	<30	<40	<15
Sensitivity	600 nA/ppm at 2 ppm	320 to 480 nA/ppm	45 nA/ppm ± 15 nA/ppm	550 ± 150 nA/ppm	0.4 ± 0.1 µA/ppm	n/a
Linearity	<±1 ppm	linear across range	<5% full scale	linear	linear	linear
Dynamic range	max. 50 ppm	max. 1000 ppm	n/a	1500 ppm	max. 500 ppm	max. 200 ppm
Power requirements	ISB, 1 mA	300 mV	n/a	300 mV	-	n/a
Dimensions (mm)	32 × 16.5	20 × 16.6	n/a	31.5 × 15.5	20.4 × 21	20.4 × 41
Weight (g)	<13	5 approx.	n/a	n/a	11	32
Environmental limits	−30 to 40°C; 80 to 120 kPa and 15% to 85% RH	−20 to 50°C; 90 to 110 kPa and 15% to 90% RH	−15 to 40°C; 20% to 90% RH	−30 to 50°C; 80 to 120 kPa and 15% to 90% RH	−20 to 50°C; 90 to 110 kPa and 10% to 90% RH	−40 to 50°C, 15% to 90% RH
Lifetime	>12 months until 50% of original	2 years	>1 year	>12 months	2 years in air	3 years in air

^l^ Alphasense Ltd. (2019): NO-B4 Nitric Oxide Sensor 4-Electrode. Technical Specification. Alphasense Ltd. Available online at http://www.alphasense.com/WEB1213/wp-content/uploads/2019/09/NO-B4.pdf, updated on July 2019, checked on 24 July 2020. ^m^ SGX Sensortech (2009): EC4-250-NO Datasheet. Nitric Oxide Electrochemical Sensor. SGX Sensortech(IS) Ltd. Available online at https://www.sgxsensortech.com/content/uploads/2014/07/EC4-250-NO1.pdf, updated on 3 October 2009, checked on 24 July 2020. ^n^ City Technology Limited (2011): Nitric Oxide Sensor Sensoric NO 3E 100. Product Data Sheet. City Technology Ltd. Available online at https://www.citytech.com/en-gb/PDF-Datasheets/no3e100.pdf, updated on November 2011, checked on 24 July 2020. ^o^ DD Scientific Limited (2019): GS+7NO Nitric Oxide Sensor (NO). Product Data Sheet. DD Scientific Ltd. Available online at http://www.ddscientific.com/uploads/5/7/1/3/57136893/gs_7no_datasheet__iss_2.pdf, updated on May 2019, checked on 24 July 2020. ^p^ PROSENSE: 4NO-250 Electrochemical NO Sensor. Product Data Sheet. ProSense Technologies Co, Ltd. Available online at http://www.szprosense.com/upfile/4NO-250EN.pdf, checked on 25 July 2020. ^q^ Membrapor AG (2020): Nitric Oxide Gas Sensor NO/S-100. Specification Sheet. Membrapor AG. Available online at http://www.membrapor.ch/sheet/Nitric-Oxide-Gas-Sensor-NO-S-100.pdf, updated on April 2020, checked on 31 July 2020.

**Table A4:** Specifications of some of the low-cost O_3_ sensors available in the market.

Company	Alphasense (UK) ^r^	Winsen (China) ^b^	City Technology (UK) ^d^	Membrapor (FR) ^s^	Ozone Solutions ^t^
**Model**	OX-B431	ZE12	A3OZ EnviroceL	CairClip (Membrapor)	Portable ozone detector S-300 EOZ
**Sensor technology**	Electrochemical	Electrochemical	Electrochemical	Electrochemical	Electrochemical
**Price in Euro**	~250	~320	~350	~200	~900
**Range**	0-20 ppm		0-10 ppm	0-250 ppm	0-10 ppm
**Resolution**	15 ppb	<10ppb	20 ppb (at 20 °C)	20 ppb	10 ppb
**Response time t_90_ in (s)**	<45 from zero to 1 ppm	<30	<40	>1	5
**Linearity**	<±0.5 ppm	n/a	linear	n/a	n/a
**Dynamic range**	max. 50 ppm	n/a	max. 100 ppm	max. 50 ppm	n/a
**Data log**	Analog	DAC, UART	n/a	USB, UART, Analog	n/a
**Power requirements**	5W, ISB, 1 mA	DC 5.0V ± 0.1 V	n/a	5V DC/200 mA	12.6 VDC
**Dimensions (mm)**	32 × 16.5	39 × 44	42.5 × 17.8	32 × 62	195 × 122 × 54
**Weight (g)**	<13	75	22	55	450
**Environmental limits**	−30 to 40°C; 80 to 120 kPa and 15% to 85% RH	−20 to 50°C; 15% to 90% RH	−20 to 50°C; atmospheric ±10%, 15% to 90% RH	−20 to 40°C; 101 ±20 kPa, 10% to 90% RH	−5 to 50°C; 5% to 95% RH
**Lifetime**	>12 months until 50% of original	2 years	2 years	1 year	n/a

^r^ Alphasense Ltd. (2019): OX-B431 Oxidising Gas Sensor Ozone +Nitrogen Dioxide 4-Electrode. Technical Specification. Alphasense Ltd. Available online at http://www.alphasense.com/WEB1213/wp-content/uploads/2019/09/OX-B431.pdf, updated on July 2019, checked on 24 July 2020. ^b^ Zhengzhou Winsen Electronics Technology Co., Ltd. (2016): Electrochemical Gas Detection Module. User’s Manual V1.2—Model: ZE12. Zhengzhou Winsen Electronics Technology Co., Ltd. Available online at https://www.winsen-sensor.com/d/files/PDF/Gas%20Sensor%20Module/Industrial%20Application%20Gas%20Sensor%20Module/ze12-electrochemical-module-manualv1_5.pdf, checked on 24 July 2020. ^d^ City Technology Limited (2001): Ozone/Nitrogen dioxide EnviroceL^®^ Specification. With assistance of Shawcity Limited. City Technology Ltd. Available online at https://www.shawcity.co.uk/documents/SensorPDFs2017/General-air-COA3OZ-EnviroceL.pdf, updated on 10 January 2001, checked on 24 July 2020. ^s^ Cairpol: Sensor-based air quality monitoring micro-stations. Ozone. Cairpol. Available online at http://cairpol.com/wp-content/uploads/2017/04/Cairpol_real_time_pollution_monitoring_sensors_EN_1116.pdf, checked on 24 July 2020. ^t^ Ozone Solutions (2020): S-300: Portable Ozone Detector with Relay Outputs. Ozone Solutions. Available online at https://ozonesolutions.com/s-300-portable-ozone-detector-with-relay-outputs/, checked on 24 July 2020.
